# CheckV assesses the quality and completeness of metagenome-assembled viral genomes

**DOI:** 10.1038/s41587-020-00774-7

**Published:** 2020-12-21

**Authors:** Stephen Nayfach, Antonio Pedro Camargo, Frederik Schulz, Emiley Eloe-Fadrosh, Simon Roux, Nikos C. Kyrpides

**Affiliations:** 1 US Department of Energy Joint Genome Institute, Lawrence Berkeley National Laboratory, Berkeley, CA USA; 2 Department of Genetics, Evolution, Microbiology and Immunology, Institute of Biology, University of Campinas, Campinas, Brazil

**Keywords:** Genome informatics, Metagenomics

## Abstract

Millions of new viral sequences have been identified from metagenomes, but the quality and completeness of these sequences vary considerably. Here we present CheckV, an automated pipeline for identifying closed viral genomes, estimating the completeness of genome fragments and removing flanking host regions from integrated proviruses. CheckV estimates completeness by comparing sequences with a large database of complete viral genomes, including 76,262 identified from a systematic search of publicly available metagenomes, metatranscriptomes and metaviromes. After validation on mock datasets and comparison to existing methods, we applied CheckV to large and diverse collections of metagenome-assembled viral sequences, including IMG/VR and the Global Ocean Virome. This revealed 44,652 high-quality viral genomes (that is, >90% complete), although the vast majority of sequences were small fragments, which highlights the challenge of assembling viral genomes from short-read metagenomes. Additionally, we found that removal of host contamination substantially improved the accurate identification of auxiliary metabolic genes and interpretation of viral-encoded functions.

## Abstract (web summary)

The quality of viral genomes assembled from metagenome data is assessed by CheckV.

## Main

Viruses are the most abundant biological entity on Earth, infect every domain of life and are broadly recognized as key regulators of microbial communities and processes^1–4^. However, it is estimated that only a limited fraction of the viral diversity on Earth can be cultivated and studied under laboratory conditions^5^. For this reason, scientists have turned to metagenomic sequencing to recover and study the genomes of uncultivated viruses^6–8^. Typically, DNA or RNA is extracted from an environmental sample, fragmented and then sequenced, generating millions of short reads that are assembled into contigs. Metagenomic viral contigs are then identified using computational tools and algorithms that use a variety of viral-specific sequence features and signatures^9–11^. In contrast to bacteria and archaea, many viral genomes are sufficiently small that they can be recovered by a single metagenomic contig^5–7^ or a single long read from Nanopore^12,13^ or PacBio technologies^14^. However, metagenome binning may be required for viruses with exceptionally large genomes, such as giant viruses^15^.

Assembly of viruses from metagenomes is challenging^16^ and the completeness of assembled contigs can vary widely, ranging from short fragments to complete or near-complete genomes^17^. Small genome fragments may adversely affect downstream analyses including estimation of viral diversity, host prediction or identification of core genes within viral lineages. Viral contigs can also be derived from integrated proviruses, in which case the viral sequence may be flanked on one or both sides by regions originating from the host genome. Host contamination also adversely affects downstream analyses^18^, especially the estimation of viral genome size, characterization of viral gene content and identification of viral-encoded metabolic genes.

For bacteria and archaea, genome quality can now be readily determined. The most widely adopted method, CheckM, estimates genome completeness and contamination based on the presence and copy number of widely distributed, single-copy marker genes^19^. Because viruses lack widely distributed marker genes, the most commonly used approach with regard to completeness is to apply a uniform length threshold (for example 5 or 10 kb) and analyze all viral contigs longer than this^5–8^. However, this ‘one-size-fits-all’ approach fails to account for the large variability in viral genome size, ranging from 2 kb in *Circoviridae*^19^ up to 2.5 megabase pairs (Mb) in *Megaviridae*^20^, and thus gathers sequences representing a broad range of genome completeness. Complete, circular genomes can be identified from the presence of direct terminal repeats^5–8^ and sometimes from mapping of paired-end sequencing reads^21^, but tend to be rare. VIBRANT^11^ and viralComplete^22^ are two recently published tools utilized to address these problems: VIBRANT categorizes sequences into quality tiers based on circularity and the presence of viral hallmark proteins, as well as nucleotide replication proteins, while viralComplete estimates completeness based on affiliation to known viruses from NCBI RefSeq.

With regard to host contamination on proviruses, existing approaches either remove viral contigs containing a high fraction of microbial genes^5^ or predict host–virus boundaries^10,11,23,24^. The former approach allows for a small number of microbial genes while the latter may fail to identify a host region or misidentify the true boundary. Other approaches detect viral signatures, but do not explicitly account for the presence of microbial regions^9^. With the diversity of available viral prediction pipelines and protocols, there is a need for a standalone tool to ensure that viral contigs do not contain contamination, and to remove it when present.

Here we present CheckV, a tool used for automatic estimation of genome completeness and host contamination for single-contig viral genomes. Based on benchmarking, we show that CheckV is computationally efficient and considerably more accurate than existing approaches. By collecting an extended database of complete viral genomes from both isolates and environmental samples, CheckV was able to estimate completeness for the vast majority of viral contigs in the IMG/VR database, illustrating its broad applicability to newly assembled genomes across viral taxa from Earth’s biomes. In addition, CheckV compares gene content between adjacent windows along each sequence to identify putative host contamination stemming from the assembly of integrated proviruses. Application to the IMG/VR database revealed that this type of contamination is rare but could easily lead to misinterpretation of viral genome size and viral-encoded metabolic genes.

## Results

### A framework for assessing the quality of single-contig viral genomes

**Fig. 1:**
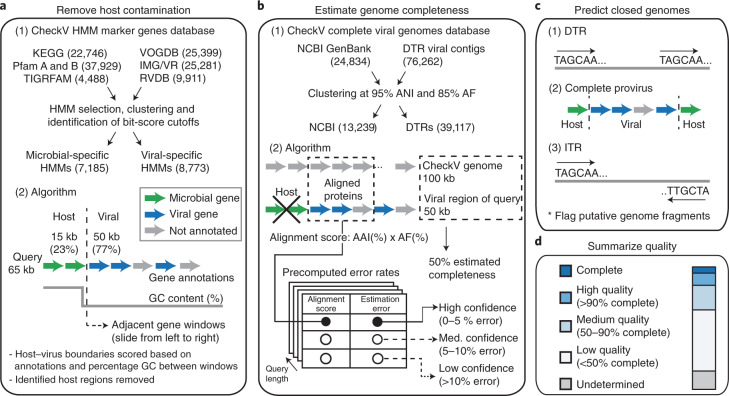
A framework for assessing the quality of single-contig viral genomes. **a**–**c**, CheckV estimates the quality of viral genomes from metagenomes in four main steps. **a**, First, CheckV identifies and removes nonviral regions on proviruses using an algorithm that leverages gene annotations and GC content. **b**, CheckV estimates genome completeness based on comparison with a large database of complete viral genomes derived from NCBI GenBank and environmental samples, and reports a confidence level for the estimate. **c**, Closed genomes are identified based on either direct terminal repeats, flanking host regions on proviruses or inverted terminal repeats. When possible, these predictions are cross-referenced with the completeness estimates obtained in **b**. Unsupported predictions are flagged as genome fragments. **d**, Sequences are then assigned to one of five different quality tiers based on their estimated completeness.

CheckV is a fully automated, command-line tool used for assessing the quality of single-contig viral genomes. It is organized into three modules which identify and remove host contamination on proviruses (Fig. 1a), estimate completeness for genome fragments (Fig. 1b) and predict closed genomes based on terminal repeats and flanking host regions (Fig. 1c). Based on these results, the program classifies each sequence into one of five quality tiers (Fig. 1d)—complete, high quality (>90% completeness), medium quality (50–90% completeness), low quality (0–50% completeness) or undetermined quality (no completeness estimate available)—which are consistent with and expand upon the Minimum Information about an Uncultivated Virus Genome (MIUViG) standards^17^. Because host contamination is easily removed, it is not factored into these quality tiers. At present, CheckV is not optimally suited for multi-contig viral genomes (for example, those identified from metagenome binning), which may contain contamination from other viruses or cellular organisms.

**Extended Data Fig. 1:**
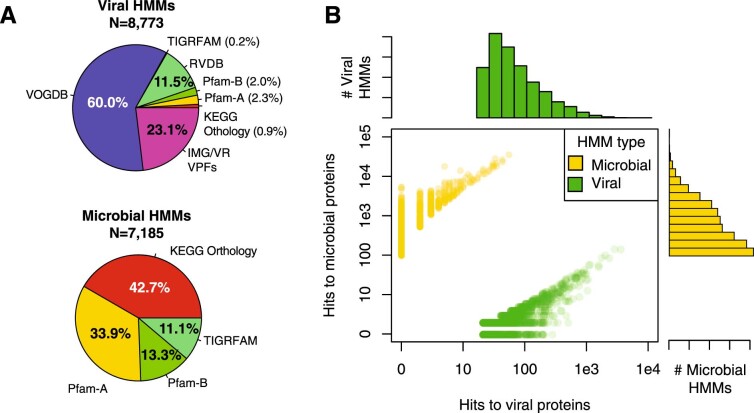
CheckV database of viral- and microbial-specific HMMs. **a**) Non-redundant viral and microbial HMMs were selected from seven reference databases. **b**) The distribution of the number of hits to viral and microbial proteins for the selected HMMs shown in panel A.

In the first step, CheckV identifies and removes nonviral regions on the edges of contigs, which can occur on assembled proviruses (Fig. 1a and Methods). Genes are first annotated as either viral or microbial (that is, from bacteria or archaea) based on comparison to a large database of 15,958 profile hidden Markov models (HMMs) (Extended Data Fig. 1 and Supplementary Table 1). We selected these HMMs from seven reference databases using three main criteria: high specificity to either viral or microbial proteins, commonly occurring in either viral or microbial genomes and nonredundant compared to other HMMs. Starting at the 5' edge of the contig, CheckV compares the genes between a pair of adjacent windows to identify host–virus boundaries characterized by a large difference in gene content (that is, microbial versus viral) and/or nucleotide composition. We optimized this approach to detect flanking host regions sensitively and specifically, even those containing just a few genes.

**Extended Data Fig. 2:**
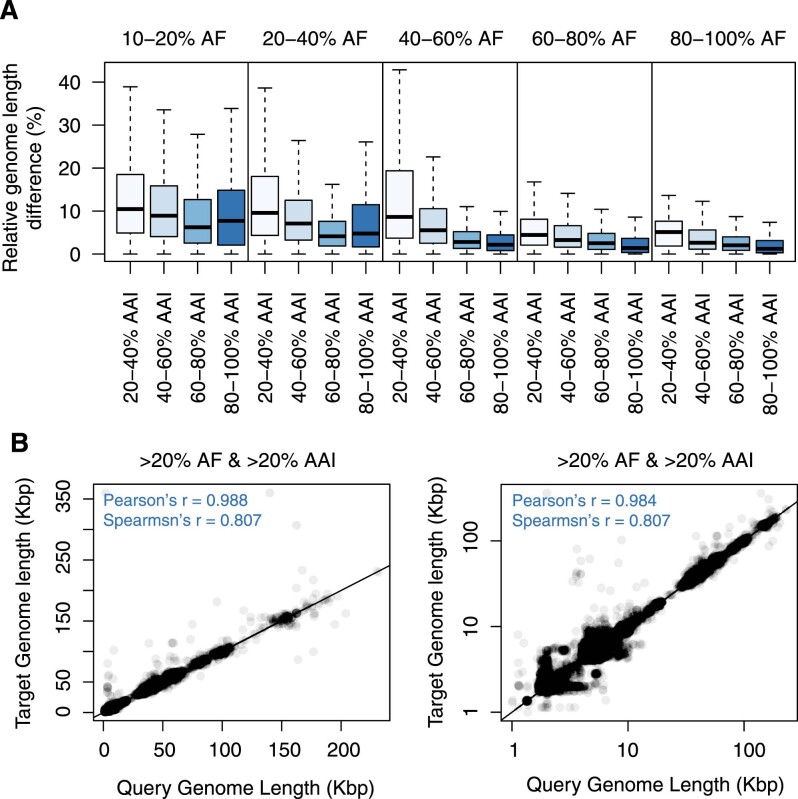
Variation in genome size between viruses. The relatedness between all CheckV reference genomes was estimated based on their average amino acid identity (AAI) and alignment fraction (AF). For box plots, the middle line denotes the median, the box denotes the interquartile range (IQR), and the whiskers denote 1.5× the IQR. **a**) The relative difference in genome length for viruses with varying degrees of relatedness. **b**) Scatterplots showing genome sizes between related viruses. The right panel shows genome sizes on a log10 scale.

In the second step, CheckV estimates the expected genome length of contigs based on the average amino acid identity (AAI) to a database of complete viral genomes from NCBI and environmental samples (Fig. 1b and Methods). The expected genome length is then used to estimate completeness as a simple ratio of lengths, similar to previous approaches^22,25,26^. In contrast to bacteria and archaea, genome length is relatively invariant among related viruses (particularly at the family or genus rank^17^), which may be a result of conserved structural features or capsid size. For example, CheckV reference genomes differed by only 12.5% in length on average (interquartile range (IQR) = 4.2–16.7%) for viruses displaying just 30–40% AAI (Extended Data Fig. 2).

Highly novel viruses may not display sufficient similarity to CheckV reference genomes for accurate estimation of completeness. To address this, CheckV reports a confidence level for each AAI-based estimate according to the expected relative unsigned error rate: high confidence (0–5% error), medium confidence (5–10% error) or low confidence (>10% error). When AAI-based completeness cannot be accurately determined (>10% error), CheckV implements a secondary approach in which the contig length is compared with that of reference genomes that are annotated by the same viral HMMs. Using this information, CheckV reports the range of completeness values corresponding to the fifth and 95th percentiles from the distribution of reference genome lengths (for example, 35–60% completeness). Compared to the AAI approach, the HMM approach can be more sensitive but is not as precise since it reports a range rather than a specific point estimate. Lastly, we designed CheckV so that its database can be readily updated to incorporate novel viral genomes as they are released in public databases (for example, NCBI GenBank and IMG/VR) or discovered from new metagenomics studies.

In the final step, CheckV predicts closed genomes based on three established approaches: direct terminal repeats (DTRs), inverted terminal repeats (ITRs) and integrated proviruses^17^. DTRs and ITRs are identified based on a repeated sequence of at least 20 base pairs (bp) at the start and end of the contig. While DTRs can play a role in genome integration^27^, they often result from assembly of short reads from a circular genome^28^ or a linear genome replicated by a mechanism involving a concatemer intermediary^29^. Pairwise alignment of DTR contigs from closely related viruses can be used to determine whether genomes have been circularly permuted^12^. Inverted terminal repeats are a hallmark of transposons^30^ but have also been observed in complete viral genomes^31^ and phages^32^. Lastly, complete proviruses are identified by a viral region flanked by host DNA on both sides and are commonly found in microbial (meta)genomes^10,11,23,24^. While these are well-established approaches, false positives have also been observed^33^ and so, to mitigate this, CheckV reports a confidence level for putative complete genomes based on the estimated completeness from the AAI- or HMM-based approaches: high confidence (≥90% completeness), medium confidence (80–90% completeness) or low confidence (<80% completeness).

### An expanded database of complete viral genomes from Earth’s biomes

**Fig. 2:**
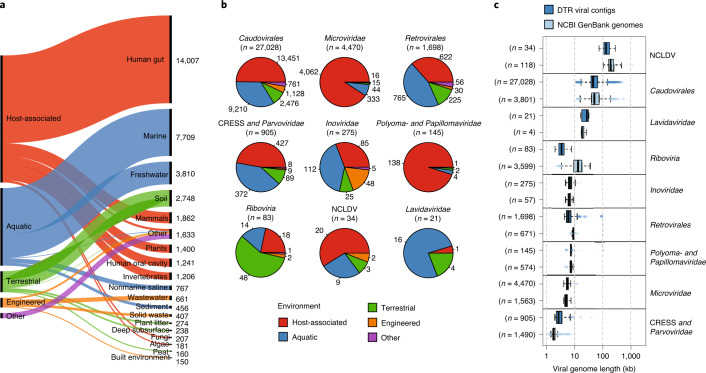
An expanded reference database of environmentally diverse complete viral genomes. **a**–**c**, The 76,262 complete viral genomes were identified from publicly available metagenomes, metatranscriptomes and viromes based on the presence of a DTR and were clustered into 39,117 nonredundant genomes at 95% ANI. **a**, The nonredundant genomes are derived from diverse human-associated and environmental habitats. Habitats are based on the Genomes OnLine Database ontology^47^, and visualization was created using RAWgraphs^48^. **b**, The 39,117 genomes were taxonomically annotated based on clade-specific marker genes from the VOG database. **c**, Comparison of sequence length between GenBank genomes and DTR contigs. For box plots, the middle line denotes the median, the box denotes IQR and the whiskers denote 1.5× IQR. NCLDV, nucleocytoplasmic large DNA virus.

We initially formed the CheckV genome database using 24,834 viral genomes from NCBI GenBank^34^ (Supplementary Table 2). However, uncultivated identified viruses commonly display little to no similarity to reference databases^5^. To mitigate this issue and expand the diversity of the database, we used CheckV to perform a systematic search for metagenomic viral contigs with DTRs (DTR contigs) from >14.4 billion contigs (9.7 terabase pairs) derived from publicly available and environmentally diverse metagenomes, metatranscriptomes and metaviromes downloaded from the following: IMG/M^35^, MGnify^36^ and recently published studies of the human microbiome^37–39^ and ocean virome^6^ (Fig. 2 and Methods). We exclusively used DTRs to identify complete genomes because this is the most well-established approach^5–8^.

**Extended Data Fig. 3:**
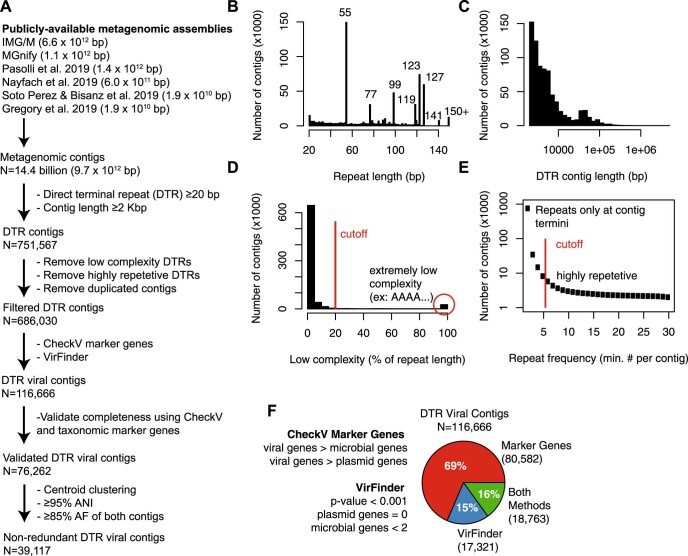
Identification of DTR viral contigs. **a**) Publicly available metagenomes were systematically mined for 76,262 DTR viral contigs, resulting in 39,117 non-redundant contigs after de-replication at 95% ANI over 85% the length of both sequences. **b**–**e**) Summary statistics across the 751,567 DTR contigs before filtering. **b**) Distribution of the length of direct terminal repeats (DTRs). A considerable number of DTRs occur at specific lengths (for example 55, 77, 99 bp). These odd-numbered lengths likely correspond with k-mer lengths utilized by various metagenomic assembly tools. When faced with assembling reads from a circular template, they appear to break the contig in a random location and leave behind a repeated sequence at the start and end of the contig equal to the k-mer length. **c**) The length (log scale) of all DTR contigs. **d**) A small number of contigs are likely false positives due to a low complexity repeat (for example AAAAAA…) or **e**) a highly repetitive repeat (that is occurring not just at termini). **f**) After removing potentially spurious complete genomes, the DTR contigs were screened for viral signatures, revealing 116,666 viral contigs. These were identified using a combination of CheckV’s marker genes, plasmid genes from recent publications, and VirFinder.

Using this approach, we identified 76,262 DTR contigs after carefully filtering out potential false positives and verifying completeness (Extended Data Fig. 3 and Supplementary Table 3). These were subsequently dereplicated to 39,117 sequences at 95% average nucleotide identity (ANI) over 85% of the length of both sequences (Supplementary Table 4). DTR contigs were found in diverse environments including the human gut (35.8%), marine (19.7%), freshwater (9.7%) and soils (7.0%) and were derived from major clades of DNA viruses, including *Caudovirales* (69.1%), *Microviridae* (11.4%) and CRESS viruses (2.3%) (Fig. 2a,b). DTR contigs were also identified for retroviruses (*Retrovirales*, *n* = 1,698) and RNA viruses (*Riboviria*, *n* = 83), which were further confirmed through identification of marker genes (for example, *RdRp*) and association with known viral families (Supplementary Information).

Next, we compared the 76,262 DTR contigs to the 24,834 GenBank references and dereplicated all sequences again at 95% ANI, resulting in 52,141 clusters. Overall, the addition of DTR contigs resulted in a 294% increase in the number of viral clusters, which was particularly pronounced for the order *Caudovirales* (611% increase) (Fig. 2b). In contrast, GenBank genomes had improved representation of other viral clades, including RNA viruses from the *Riboviria* realm (Supplementary Table 5). For most viral clades, the sizes of DTR contigs and GenBank genomes were consistent, indicating no systematic artifacts in our data (Fig. 2b). One interesting exception was for segmented RNA viruses (*Riboviria* and *Retrovirales*), in which the DTR contigs tended to be smaller than the GenBank references, suggesting that they either represent a single genome segment or cover only a subset of the diversity within these large groups.

### Accurate estimation of genome completeness for novel viruses

**Fig. 3:**
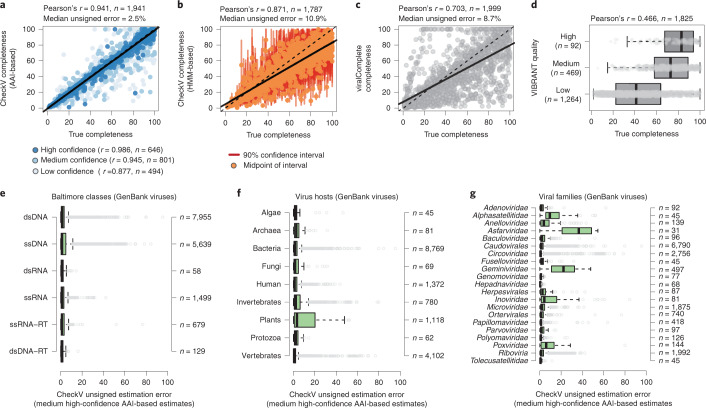
Benchmarking completeness estimation for CheckV and existing tools. **a**–**d**, Benchmarking completeness for a mock dataset containing fragments from 2,000 complete genomes derived from IMG/VR. Dashed lines represent the line of equality while solid lines indicate best fit. Completeness estimates above 100% were set to 100%. **a**, CheckV-estimated completeness using the AAI-based approach. Point color depth indicates estimation confidence level. **b**, CheckV-estimated completeness using the HMM-based approach. Red vertical lines indicate the 90% confidence interval of estimated completeness while points indicate the midpoint of that interval. **c**, Completeness as estimated by viralComplete, based on the ratio of the contig length to the length of the classified reference genome. **d**, VIBRANT quality tiers. **e–g**, Benchmarking CheckV completeness for genome fragments derived from NCBI GenBank genomes. Estimation error is shown for viruses according to their Baltimore classification (**e**), cellular host (**f**) and viral family (**g**). Only medium- and high-confidence AAI-based estimates are shown. Viral categories representing at least 30 viruses are indicated on the vertical axes. For box plots, the middle line denotes the median, the box denotes the IQR and the whiskers denote 1.5× IQR. RT, reverse transcribing; ssDNA, single-stranded DNA.

Having developed the CheckV pipeline and databases, we next benchmarked its accuracy. To evaluate genome completeness, we generated a mock dataset containing fragments from 2,000 uncultivated, complete viral genomes derived from IMG/VR (Supplementary Table 6). We first estimated completeness using the AAI-based approach after removal of all closely related sequences from the CheckV database (>95% AAI). This revealed a strong correlation with expected values (Pearson’s *r* = 0.941), low overall error (2.5% median unsigned error (MUE), IQR = 0.91–5.8%) and estimates for 97% of sequences (Fig. 3a). As expected, accuracy varied according to the confidence level, with best performance for high- and medium-confidence estimates (Pearson’s *r* = 0.986 and 0.945, respectively). Next we applied the HMM-based approach, which yielded estimated completeness for 89.4% of fragments and 94.5% >5 kb. While HMM-based estimates were considerably less accurate (Pearson’s *r* = 0.871), the lower bound of the estimated range was consistently below the expected value (96.6% of the time; Fig. 3b). This indicates that the HMM-based approach can be useful for accurately obtaining a conservative lower bound on genome completeness, particularly for novel viruses that display low AAI to CheckV reference genomes.

For comparison, we applied viralComplete^22^ and VIBRANT^11^ to the mock dataset (Fig. 3c,d and Supplementary Table 6). viralComplete estimates completeness based on affiliation to viruses from the NCBI RefSeq database, while VIBRANT classifies sequences into quality tiers based on gene content and the presence of DTRs. VIBRANT’s quality tiers were only weakly correlated with true completeness (Pearson’s *r* = 0.466), with a majority of near-complete genomes (>90% complete) classified as either low or medium quality (183/227, 80.6%). viralComplete showed much better performance (Pearson’s *r* = 0.703), but still displayed high error for a considerable number of sequences (MUE = 8.71%, IQR = 3.13–21.76%).

Based on the presence of clade-specific marker genes, we determined that most sequences from the mock dataset belonged to the *Caudovirales* order of double-stranded DNA phages. To evaluate performance for other types of viruses, we applied CheckV to viral genome fragments from NCBI GenBank after removal of closely related genomes from the CheckV database (Supplementary Table 7). Using the AAI-based approach (excluding low-confidence estimates), completeness was accurately estimated overall (MUE = 1.33%, IQR = 0.45–3.57%) including for viruses from different Baltimore classes, those infecting various hosts and those from different families (Fig. 3e–g). A few viral groups were problematic, including single-stranded DNA plant viruses from the family *Geminiviridae* (MUE = 22.2%), where CheckV did a poor job of distinguishing between monopartite and segmented genomes, and the *Asfarviridae* family (170–190 kb), where CheckV identified the giant viruses Pacmanvirus (395.4 kb) and Kaumoebavirus (350.7 kb) as nearest relatives in the database.

**Extended Data Fig. 4:**
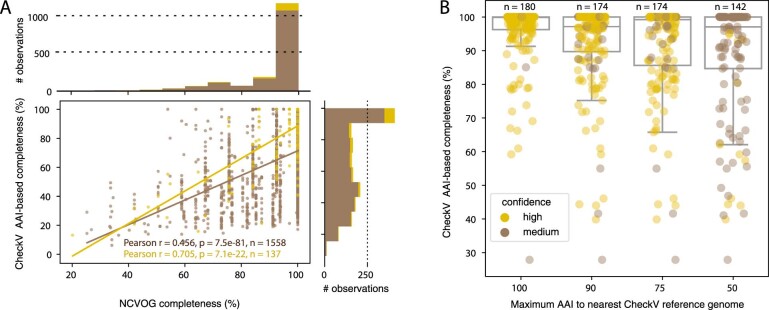
Evaluation of CheckV completeness estimates for giant virus metagenome assembled genomes (GVMAGs) and nucleocytoplasmic large DNA virus (NCLDV) isolate genomes. **a**) Correlation between CheckV completeness (medium and high confidence AAI-based estimates) versus low-copy number NCVOG completeness estimates for 2,074 GVMAGs. Points in the scatter plot indicate GVMAGs that had a high (yellow) or medium (brown) confidence CheckV completeness estimate. Histograms indicate the total number of GVMAGs across different completeness intervals for both approaches. GVMAGs with undetermined or low-confidence CheckV estimates were excluded from the analysis (n = 379). Completeness estimates above 100% were set to 100%. *P values* were calculated using a two-sided Pearson regression. **b**) CheckV completeness estimates for 182 NCLDV isolate genomes after excluding closely related CheckV reference genomes. The maximum amino acid identity between CheckV references and NCLDV isolate genomes is indicated by the plot labels. Center lines of box plots represent the median, bounds of boxes the lower and upper quartile, whiskers extend to points that lie within 1.5 interquartile range of the lower and upper quartile. Each data point represents the completeness estimate for a NCLDV reference genome, where the color indicates the CheckV confidence level. Completeness estimates above 100% were set to 100%.

While CheckV is not ideally suited for viral bins, we evaluated its performance on a recent dataset of 2,074 giant virus metagenome-assembled genomes (GVMAGs)^15^ in which Schulz et al. estimated genome completeness based on 20 low-copy number nucleocytoplasmic virus orthologous groups (NCVOGs)^15^ (Extended Data Fig. 4a). Using the AAI-based approach, CheckV estimated completeness for 6.6, 75.1 and 18.3% of GVMAGs at the high-, medium- and low-confidence levels, respectively. Overall, CheckV completeness estimates were correlated with those from the NCVOG approach (Pearson’s *r* = 0.451), but particularly for high-confidence CheckV estimates (Pearson’s *r* = 0.705). Similar results were observed based on an analysis of genome fragments from nucleocytoplasmic large DNA virus isolates^40^ (Extended Data Fig. 4b). These correlations imply that CheckV gave broadly similar results compared to those of Schulz et al.^15^, and may be suitable for evaluation of the completeness of certain metagenome-assembled giant virus genomes.

### Accurate identification of host regions on proviruses

**Fig. 4:**
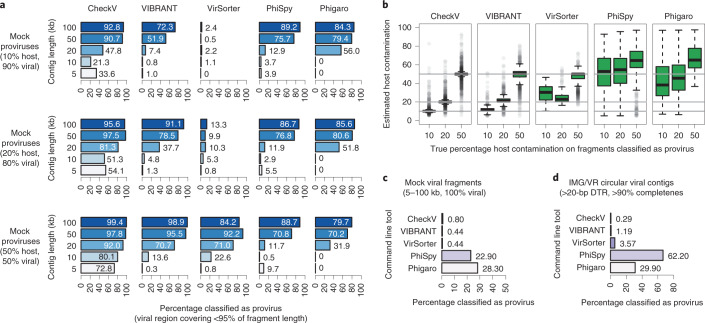
Sensitivity and specificity of predicting host regions on viral contigs. **a**–**c**, Mock proviruses were generated for 382 paired bacteriophage–host pairs. Random genome fragments were extracted from mock proviruses at various lengths and levels of host contamination and used as input to CheckV and other tools. A fragment was classified by a tool as a provirus if it contained a predicted viral region that covered <95% of the fragment length. **a**, Sensitivity of CheckV and other tools in detecting host contamination on proviral fragments with at least 10% host contamination (*n* = 4,593). **b**, Estimated contamination versus true contamination for correctly classified provirus fragments. For box plots, the middle line denotes the median, the box denotes the IQR and the whiskers denote 1.5× IQR. **c**, To determine specificity, CheckV and other tools were used to predict host regions on entirely viral fragments (*n* = 1,367). **d**, CheckV and other tools were used to predict host regions on circular viral contigs from IMG/VR (*n* = 1,345).

Next, we evaluated CheckV’s accuracy in detecting host contamination on provirus sequences (Supplementary Table 8). To generate mock proviruses, we identified 382 bacteriophages from NCBI and paired them with their bacterial and archaeal hosts from the Genome Taxonomy Database (GTDB)^41^. After inserting each phage at a random location in its host genome, we extracted genomic fragments varying in both length (5–50 kb) and amount of flanking host sequence (10–50%). Overall, CheckV detected the presence of host regions on 69.0% of mock proviruses and 88.3% for contigs ≥20 kb (Fig. 4a). The length of host regions was also accurately estimated (Fig. 4b), with a median unsigned error of only 0.6% (IQR = 0.16–2.2%). As a negative control, we applied CheckV to genomic fragments that were entirely viral (that is, no flanking host region). Only 0.80% of these sequences were classified as proviruses, indicating that CheckV has a low provirus false-positive rate (Fig. 4c). Similar results were observed when we applied CheckV to complete uncultivated viral genomes from IMG/VR (Fig. 4d and Supplementary Table 9).

For comparison, we evaluated four other tools to identify host–provirus boundaries, including VIBRANT^11^, VirSorter^10^, PhiSpy^23^ and Phigaro^24^. Compared to these four tools, CheckV displayed consistently higher sensitivity but, in particular, when fragments were short or host contamination was low (Fig. 4a). For example, VirSorter detected only 1.2% of proviruses with 10% contamination compared to 57.2% with CheckV. This implies that microbial genes at the edges of proviral contigs may be overlooked by existing tools and interpreted as viral encoded. In contrast, other tools identified host–virus boundaries on entirely viral sequences (Fig. 4c,d). For example, PhiSpy predicted nonviral regions on 22.9% of entirely viral fragments that covered 26.3% of the length of these sequences on average. This implies that truly viral regions may be discarded with existing tools and that sequences may be inadvertently classified as integrated proviruses. Apart from CheckV, VIBRANT performed optimally at identification of flanking host regions on proviruses.

Finally, we compared the computational efficiency of CheckV to existing tools. Using 16 CPUs (Intel Xeon E5–2698 v3 processors), the full CheckV pipeline was 1.6- to 11.6-fold faster than the other programs when applied to the mock dataset and required ~2 GB of memory. Using a single CPU, CheckV was still faster than VirSorter and VIBRANT but slower compared to viralComplete, PhiSpy and Phigaro (Supplementary Table 10).

### Using CheckV to identify high-quality genomes from metagenomes and viromes

To illustrate the type of results obtained with CheckV and its ability to scale to large datasets, we applied it to 735,106 viral contigs from the IMG/VR 2.0 database^25^ (Supplementary Table 11). IMG/VR contigs were previously identified from short-read metagenomic assemblies using the Earth’s Virome Protocol^5^ and a minimum contig length cutoff of 5 kb. The original samples were derived from many studies, the majority of which did not use size filtration to enrich for extracellular viral particles. Because of the sample characteristics and detection approach, this dataset is mostly composed of environmental dsDNA phages from the *Caudovirales* order and contains sequences from both lysogenic and lytic viruses.

**Extended Data Fig. 5:**
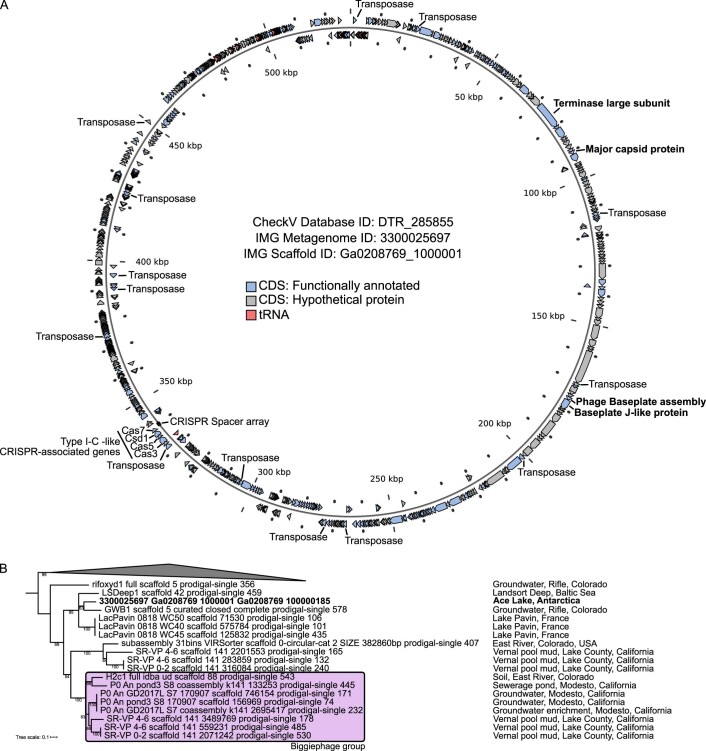
Genome map and phylogeny of the largest complete genome from IMG/VR. **a**) Genome map of 528,258 bp putative circular metagenomic contig (IMG identifier: Ga0222679_1000001), sampled from Ace Lake in Antarctica. Annotations were obtained from IMG and manual annotation of phage proteins (terminase and major capsid protein) via HHPred. **b**) TerL phylogeny of the circular metagenomic contig with the most closely related sequences from a global search for large phages.

**Extended Data Fig. 6:**
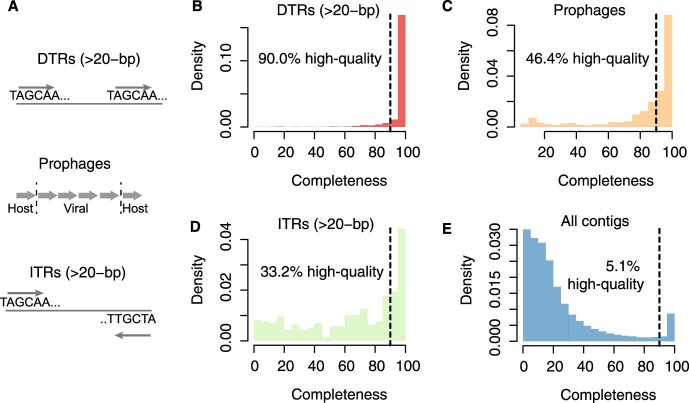
Evaluating metagenomic signatures of complete viral genomes. Putative complete viral genomes were identified from the IMG/VR database based on either A) DTRs (N = 15,211), B) proviruses with flanking host regions (N = 451), or C) ITRs (N = 624). In parallel, the completeness of IMG/VR contigs was estimated using the AAI-based approach, using only high- and medium-confidence level estimates. The histograms above show the distribution of completeness for each signature of complete genomes (high- and medium- confidence estimates only), as well as the distribution of completeness across all other IMG/VR contigs. The text indicates the percent of IMG/VR contigs in each category that are classified as high-quality (that is >90% estimated completeness).

First, we used CheckV to identify three types of complete genome from IMG/VR, including DTR contigs (*n* = 15,211), proviruses with 5' and 3' host regions (*n* = 451) and contigs with ITRs (*n* = 624). The longest DTR contig we identified was a 528,258-bp sequence from a saline lake in Antarctica estimated to be 100.0% complete and supported by paired-end reads that connected contig ends. Based on gene content and phylogeny, this sequence is probably a novel member of one of the recently defined clades of ‘huge’ phages^42^ (Supplementary Text and Extended Data Fig. 5). To validate the other potentially complete genomes, we compared the contigs to CheckV’s database of complete reference genomes based on AAI, estimated completeness (excluding low-confidence estimates) and identified high-quality assemblies (that is, >90% complete). We found that 90.0% of the DTR contigs with estimated completeness met the high-quality standard compared to only 46.4% of complete proviruses and 33.2% of ITRs (Extended Data Fig. 6). In the case of proviruses, lower estimated completeness may be due to their domestication and degradation in the host genome over time^43^ or, in rare cases, due to false positives. Nonetheless, predictions from all three methods were highly enriched in high-quality genomes compared with other contigs from IMG/VR. These results further confirm that DTRs are a good indicator of complete viral genomes most of the time^33^, but suggest that greater caution is needed when interpreting other signatures.

**Extended Data Fig. 7:**
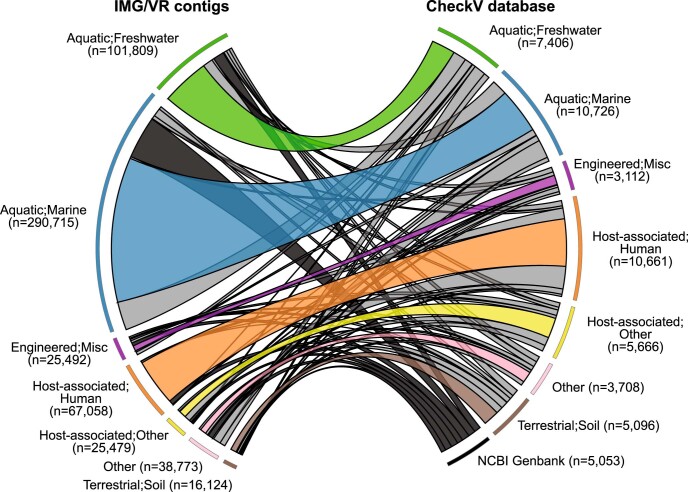
Association between IMG/VR contigs and CheckV reference genomes. IMG/VR contigs (left) are classified by the biome of their original metagenomes and connected to their top hit in the CheckV database based on amino acid identity (right). Cases in which IMG/VR contigs and CheckV references are derived from the same habitat (for example marine IMG/VR contig and marine CheckV reference) are colored by biome, while other cases are colored in grey.

**Extended Data Fig. 8:**
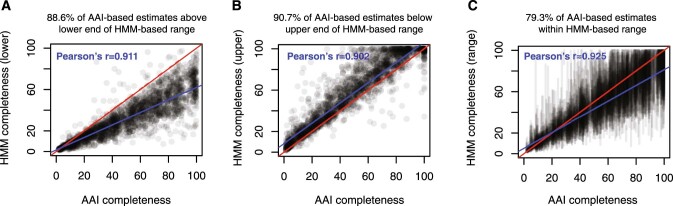
Comparison between AAI and HMM-based completeness estimates. The analysis above was based on 568,096 IMG/VR contigs with both AAI- and HMM-based completeness estimates. Note that the AAI-based approach results in a point estimate whereas the HMM-based approach results in a range representing the estimated 90% confidence interval based on the empirical distribution of reference genome lengths sharing the same HMMs. A random subset of points shown for each plot. **a**) Comparison between AAI-based estimates and the lower end of the HMM-based range. As expected, most AAI-based estimates fall above the lower end of the HMM-based range. This percentage is lower than 95% (the target value) likely due to error in AAI- and HMM-based estimates. Each point is a single contig. **b**) Comparison between AAI-based estimates and the upper end of the HMM-based range. Each point is a single contig. **c**) Comparison between AAI-based estimates and the midpoint of the HMM-based range. Each line is a single contig. For all figures, the line of identity is indicated in red while the line of best fit is indicated in blue. The Pearson correlation coefficient is shown in blue.

Next, we used CheckV to estimate completeness for the entire IMG/VR dataset, including genome fragments. Using the accurate AAI-based approach, completeness could be estimated for 80.2% of IMG/VR contigs with high or medium confidence, including 84.5% from host-associated, 83.9% from marine, 75.1% from freshwater and 70.0% from soil environments. For the majority of these contigs, the best hit in the CheckV database was a DTR sequence (*n* = 501,055, 85.0%) and was often derived from the same habitat as the IMG/VR contig (Extended Data Fig. 7). We next applied the HMM-based approach to estimate the completeness range for novel IMG/VR contigs lacking confident AAI-based estimates, increasing the percentage of contigs with estimated completeness to 97.9. AAI- and HMM-based estimates were well correlated for contigs having both predictions (Spearman’s *ρ* = 0.90), with AAI-based predictions often falling within the completeness range predicted by the HMM approach (Extended Data Fig. 8).

**Fig. 5:**
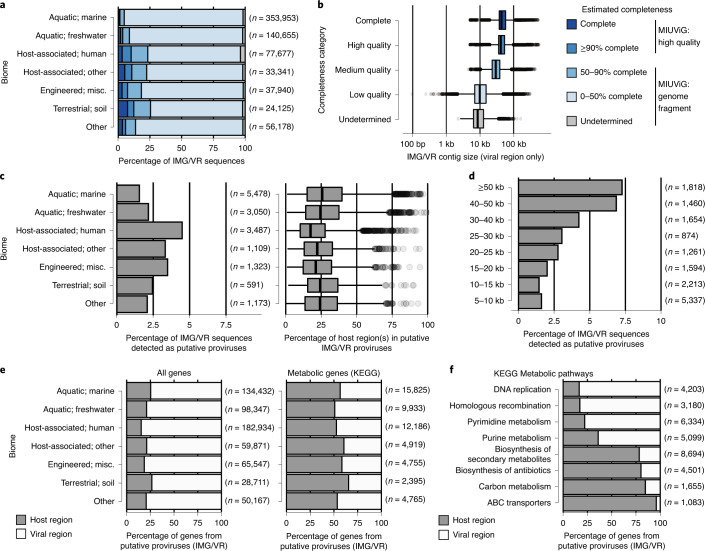
Application of CheckV to the IMG/VR database. **a**, Estimated completeness of IMG/VR contigs by biome. **b**, Distribution of IMG/VR contig length across quality tiers: complete (*n* = 13,700), high quality (*n* = 16,544), medium quality (*n* = 45,109), low quality (*n* = 634,117) and undetermined (*n* = 14,399). For proviruses, only the size of the predicted viral region was considered. **c**, Proportion of IMG/VR contigs predicted as proviruses by biome (left). Sequences predicted with >50 ambiguous bases (Ns) or potential concatemers were classified as low quality. Putative nonviral sequences in IMG/VR were not included (>5 host genes and >2× host versus viral genes). Length of the predicted host region by biome for IMG/VR contigs predicted as proviruses (right). Region length is indicated as a percentage of total contig length. **d**, Proportion of contigs predicted as proviruses by contig length. **e**, Percentage of all genes from predicted proviruses found in viral/host regions (left). Percentage of metabolic genes from predicted proviruses found in viral/host regions (right). **f**, Percentage of genes from selected KEGG pathways for predicted proviruses found in viral/host regions. For box plots, the middle line denotes the median, the box denotes the IQR and the whiskers denote 1.5× IQR. Misc., miscellaneous.

**Extended Data Fig. 9:**
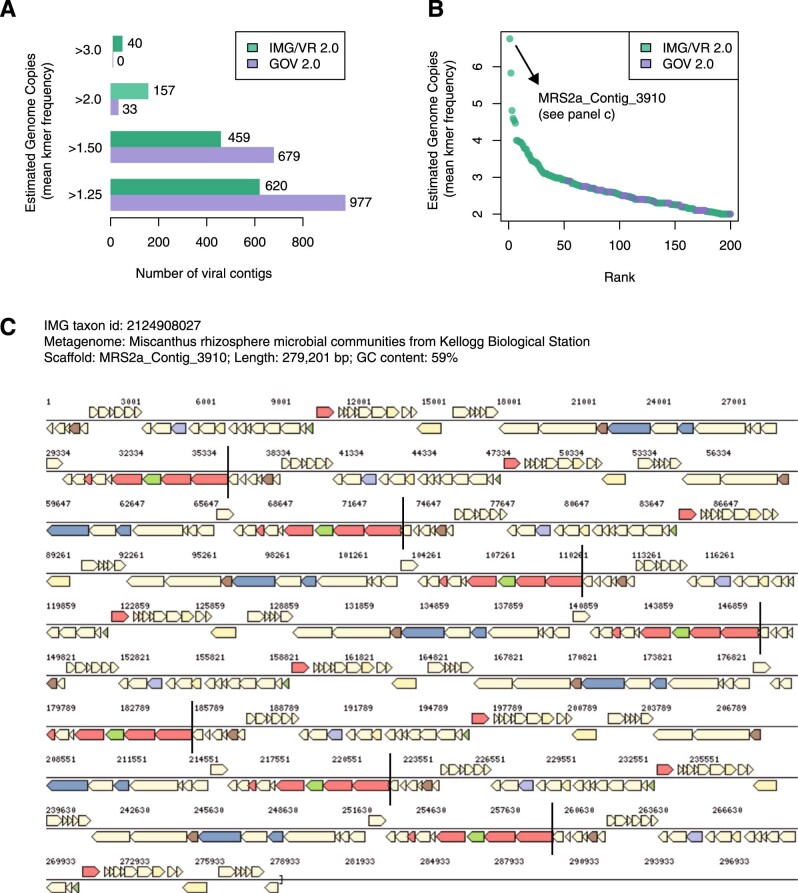
Identification of contigs with concatenated genomes in public databases. CheckV was used to quantify the average *k*-mer frequency for viral contigs in IMG/VR and the GOV. A contig that contains exactly one genome copy should have a *k*-mer frequency close to 1.0 (all kmers are unique). Contigs that contain the same repeated genome (for example concatemers) will have *k*-mer frequencies above 1.0. **a)** The number of viral contigs at different cutoff points for the *k*-mer frequency statistic. **b**) Top 100 contigs with the highest *k*-mer frequencies. **c**) An example of a 279,201 bp contig in IMG/VR with the same sequence repeated more than 7x.

We next classified IMG/VR sequences into quality tiers according to their estimated completeness, revealing 1.9% complete, 2.4% high-, 6.4% medium- and 87.3% low-quality sequences, with the remaining 2.0% of undetermined quality (Fig. 5a). Contig sizes were strongly correlated with quality tiers, with complete genomes centered at 44 kb, which is consistent with genome sizes from the order *Caudovirales* (Fig. 5b). In a small number of cases, IMG/VR contigs were considerably longer than expected (for example, 290 contigs more than twofold expected length based on AAI) and which, upon inspection of *k*-mer frequencies, revealed the same genome repeated multiple times (2.0× to 6.8×) (Extended Data Fig. 9). While these are probably artifacts of metagenomic assembly, they are easily identified and less common than previously suggested^42^.

**Extended Data Fig. 10:**
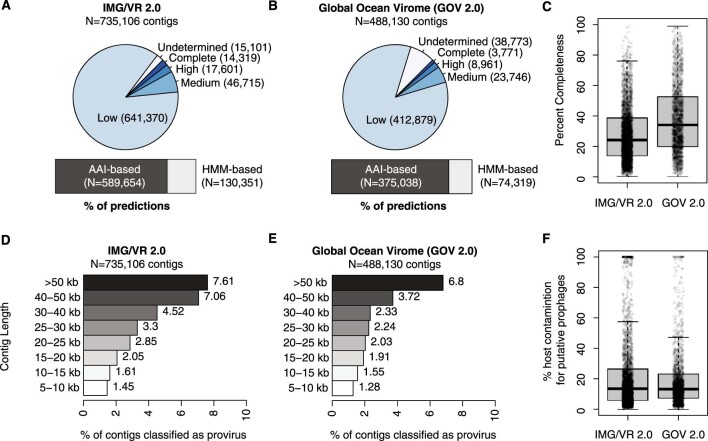
Application of CheckV to the IMG/VR 2.0 and the Global Ocean Virome 2.0 datasets. **a**) Quality tiers across viral contigs from IMG/VR 2.0 and **b**) the GOV 2.0 dataset. The bar plots indicate the % of completeness estimates made with the AAI- or HMM-based approaches. **c**) Distribution of AAI-based completeness across contigs from each dataset. **d**) Percent of contigs classified as a provirus for IMG/VR 2.0 and **e**) for GOV 2.0. **f**) Distribution of host contamination (that is percent of length derived from host regions on proviruses) across datasets. For box plots, the middle line denotes the median, the box denotes the interquartile range (IQR), and the whiskers denote 1.5× the IQR.

We also applied CheckV to the Global Ocean Virome (GOV) 2.0 dataset^6^ (Supplementary Table 12), which revealed remarkably similar patterns (Extended Data Fig. 10). Like IMG/VR, the GOV dataset contains viral contigs that are at least 5 kb but, unlike IMG/VR, the original samples were derived from the open ocean and enriched for viral particles before sequencing. We identified a combined total of 44,652 complete or high-quality genomes across both datasets, but these represented a mere 3.6% of the total number of contigs. The highly fragmented nature of sequences from IMG/VR and GOV probably reflects numerous challenges in the assembly of viruses from short-read metagenomes, including repetitive regions^12^, strain heterogeneity^13^, low-abundance viral populations^33^ and low sample biomass^44^. Long-read sequencing circumvents many of these challenges and has recently been used to obtain high-quality viral genomes without the need for metagenomic assembly^12,13^.

### Using CheckV to discriminate viral-encoded functions from host contamination

Finally, we used CheckV to identify putative proviruses from the IMG/VR database that were flanked on one or both sides by host genes. Overall, only 17,057 contigs followed this pattern (Fig. 5c) with 96.5% of host regions occurring on only one side and typically representing a minor fraction of contig length (average, 26.8%; Fig. 5d). Proviruses were detected in all biomes, although more frequently in host-associated metagenomes. Longer contigs were considerably more likely to contain a host region (Fig. 5d), which is probably explained by the higher sensitivity of CheckV for longer sequences and a greater chance of intersecting a host–provirus boundary. Supporting these predictions, the majority of long proviruses (>50 kb with >20% contamination, *n* = 783) were confirmed by either VirSorter or VIBRANT (76.8%) and contained integrases (85.2%). We also used CheckV to identify proviruses in the GOV dataset, revealing similar patterns (Extended Data Fig. 10). Together, these results confirm that the majority of IMG/VR and GOV sequences are entirely viral or encode a short, host-derived region.

Notably, even a small amount of contamination by host-derived sequences can impair downstream analyses, especially those related to the gene content and functional potential of uncultivated viruses^18^. To illustrate this potential issue, we functionally annotated the 17,057 IMG/VR proviruses using the Kyoto Encyclopedia of Genes and Genomes (KEGG) database^45^ and compared the functions of genes in host versus viral regions. Overall, host regions represented only 19.2% of the genes but 59.7% of genes assigned to a KEGG metabolic pathway (Fig. 5f). Several pathways were highly enriched in host genes, including those for biosynthesis of antibiotics, carbon metabolism and ABC transporters (Fig. 5g and Supplementary Table 12). For example, 254 provirus genes were annotated as multidrug efflux pumps or multidrug resistance proteins, but 95.3% of these were found in host regions. In contrast, KEGG pathways for recombination, mismatch repair and nucleotide biosynthesis were all highly enriched in viral regions. Without the detection of provirus boundaries provided by CheckV, it would not have been possible to discriminate true viral-encoded functions from host contamination except through manual curation, which becomes nearly impossible for large datasets like IMG/VR.

## Discussion

Here we have presented CheckV, an automated pipeline used for assessing the quality of single-contig viral genomes, along with an expanded database of complete viral genomes that we systematically identified from environmental data sources. We anticipate that CheckV will be broadly useful in future viral metagenomics studies and for reporting quality statistics required in the MIUViG checklist^17^. Estimation of completeness will be especially valuable in distinguishing near-complete genomes from short genome fragments, as these two types of sequence are associated with different limitations and biases. For example, the inclusion of small genome fragments may result in inflated estimates of viral diversity based on genome clustering due to insufficient overlap between sequences. Meanwhile, the removal of genes originating from the host genome will be critically important in reducing false positives in viral studies focusing on auxiliary metabolic genes or the discovery of novel protein families. We also expect that CheckV’s database of complete viral genomes will be a useful community resource that contains a wealth of untapped insights into novel viruses from diverse environments.

Several improvements in CheckV may be possible in the future. First, it will be important to incorporate new viral genomes as these become available, to continually expand the environmental and taxonomic diversity of the reference database. Inclusion of novel RNA viruses and eukaryotic viruses will be especially valuable, as these types of genome are currently under-represented in the database. Second, metagenomic read mapping could be used for a variety of inferences, including identification of circular contigs^21^, refinement of virus–host boundaries and determination of genome termini^46^. Third, viral MAGs (that is, derived from metagenome binning) and segmented viral genomes, which are represented by multiple sequences, pose several additional challenges not addressed here, including the presence of contamination from other viruses or cellular organisms. Finally, CheckV could be adapted to detect other artifacts, such as chimeras resulting from the assembly of closely related viruses or nonviral sequences resulting from false-positive viral predictions.

## Methods

### Database of HMMs for classification of viral and microbial genes

We selected HMMs from existing databases that could be leveraged to classify genes as either viral or microbial with high specificity. First, 125,754 HMMs were downloaded from seven databases: VOGDB (release 97, *n* = 25,399, http://vogdb.org), IMG/VR (downloaded January 2020, *n* = 25,281)^25^, RVDB (release 17, *n* = 9,911)^49^, KEGG Orthology (release 2 October 2019, *n* = 22,746)^45^, Pfam A (release 32, *n* = 17,929)^50^, Pfam B (release 27, *n* = 20,000)^51^ and TIGRFAM (release 15, *n* = 4,488)^52^. Next, we used hmmsearch v.3.1b2 (ref. ^53^) to align the HMMs versus 1,590,764 proteins from 30,903 NCBI GenBank viral genomes (downloaded 1 June 2019)^34^ and 5,749,148 proteins from 2,015 bacterial and 239 archaeal genomes from GTDB, release 89). For computational reasons, we selected a maximum of one genome per GTDB family and, when multiple genomes were available, we chose the one with the highest CheckM quality score (completeness – 5 × contamination). Additionally, we ran VIBRANT v.1.2.0 (ref. ^11^), VirSorter v.1.0.5 (ref. ^10^) and PhiSpy v.3.7.8 (ref. ^23^) using default parameters to identify and remove 590,484 viral proteins identified on proviruses in the selected GTDB genomes.

Based on the hmmsearch results, we calculated the percentage of viral and microbial genes matching each HMM at bit-score cutoffs ranging from 25 to 1,000, in increments of 5. We then selected the lowest bit-score cutoff for each HMM that resulted in a difference >100-fold between the percentage of the total viral gene set and that of the total microbial gene set matched by the HMM (that is, bit-score cutoff for which the hits were strongly enriched in either virus or microbial genes). To limit false positives, we excluded HMMs that were classified as microbial specific but were derived from primarily viral databases (VOGDB, IMG/VR, RVDB) or contained viral terms (viral, virus, virion, provirus, capsid, terminase) for HMMs from other databases. Using this approach, 114,765 HMMs were identified as either viral specific or microbial specific.

Next, we selected the maximally informative subset of HMMs to reduce the size of the database and limit CheckV computing time. First, we retained 44,415 HMMs with at least 20 viral hits or at least 100 microbial hits after applying the bit-score cutoffs. Next, we calculated the Jaccard similarity between all pairs of HMMs based on each HMMs set of gene hits. For computational efficiency, we used the ‘all_pairs’ function in the SetSimilaritySearch Python package (https://github.com/ekzhu/SetSimilaritySearch). Jaccard similarities were used as input for single-linkage clustering with a Jaccard similarity cutoff of 0.5, resulting in 15,958 nonredundant HMMs (8,773 viral specific, 7,185 microbial specific). To form the final database, we selected the HMM with the greatest number of gene hits from each cluster of HMMs.

### Identification of virus–host boundaries

Given a viral contig, CheckV predicts host–virus boundaries in three stages.

First, proteins are predicted using Prodigal v.2.6.3 (option ‘-p meta’ for metagenome mode)^54^ and compared to the 15,958 HMMs using hmmsearch. Each protein is classified as viral, microbial or unannotated according to its top-scoring hit after applying the HMM-specific bit-score cutoffs. Viral- and microbial-annotated genes are assigned a viral score of +1 and –1, respectively. Additionally, the GC content of each gene is calculated (range, 0–100).

Second, CheckV scans across the contig and quantifies differences in the viral score (that is, +1 or –1) and GC content between a pair of adjacent gene windows. The 5' gene window extends to the left contig endpoint, and the 3' gene window is sized to contain 30% of genes on the contig with no fewer than 15 genes and no more than 50 genes. The 3' window may contain fewer then 15 genes if it ends at the right contig endpoint. CheckV then computes a breakpoint score, *S*, based on the absolute difference in the average viral score, *V*, and average GC content, *G*, between genes in the 5' and 3' windows: *S* = |*V*_5'_ – *V*_3'_| + 0.02×|*G*_5'_ – *G*_3'_|. Unannotated genes are not included when calculating *V*. The value of *S* ranges from 0 to 4, given that |*V*_5'_ – *V*_3'_| and 0.02×|*G*_5'_ – *G*_3'_| both range from 0 to 2. CheckV also stores the orientation of each breakpoint (that is, host–virus or virus–host) based on the values of *V*_5′_ and *V*_3′_. These scores are computed at each intergenic position, moving from the 5' end to the 3' end of the contig.

Third, CheckV identifies breakpoints based on the following rules: *S* ≥ 1.2, ≥30% genes annotated as microbial in the host region, ≥2 microbial-annotated genes in the host region and ≥2 viral-annotated genes in the viral region. For very short contigs (fewer than ten genes), CheckV requires only one microbial-annotated gene in the host region and one viral-annotated gene in the viral region. After these filters, CheckV chooses the first encountered breakpoint with the highest score. After selecting the first breakpoint, CheckV then repeats the steps listed above to search for additional breakpoints, using the last identified breakpoint as the new starting position for the 5' gene window. The algorithm ends when no new breakpoints are found. Algorithm parameters were fine-tuned empirically based on a dataset of mock proviruses and sequences from the IMG/VR database.

### AAI-based estimation of genome completeness

Given a viral contig, CheckV estimates genome completeness in four stages. First, it performs an amino acid alignment of Prodigal-predicted protein-coding genes from the contig against the database of reference genomes using DIAMOND v.0.9.30 (ref. ^55^), with the option ‘–evalue 1e-5–query-cover 50 --subject-cover 50 -k 10000’. Based on these alignments, the following metrics are computed for the viral contig versus each reference genome: AAI: length-weighted average identity across aligned proteins; alignment fraction (AF): percentage of amino acids aligned from the query sequence; and alignment score: AAI × AF. Second, CheckV identifies the top hit in the database for the contig (that is, the reference genome with the highest alignment score) and all reference genomes with alignment scores within 50% of the top hit. The expected genome length of the viral contig, ${\hat{\it G}}$, is then estimated by taking a weighted average of the genome sizes of matched reference genomes, where the alignment scores are used as weights. Reference genome lengths are further weighted based on their source: 2.0 for isolate viruses and 1.0 for metagenome-derived viruses, which are more likely to contain assembly errors and artifacts. CheckV also reports the confidence level of this estimate (low, medium or high), which is determined based on the length of the viral contig and the alignment score to the top reference genome (see Confidence levels for AAI-based completeness estimates for the method used to estimate confidence levels). Third, CheckV estimates the genome completeness of each viral contig, ${\hat{\it C}}$, using the formula: ${\hat{\it C}} = 100 \times {\it{L}}/{\hat{\it G}}$, where *L* is the length of the viral region for proviruses, or the contig length otherwise.

### HMM-based estimation of genome completeness

An HMM-based approach was developed to estimate completeness for novel viruses that are too diverged from CheckV genomes to obtain an accurate AAI-based estimate. First, CheckV identifies viral genes on the contig based on comparison to the 8,773 viral HMMs (see ‘Identification of virus–host boundaries’ above). Each viral HMM is associated with one or more reference genomes and this information is stored in the database, as well as the coefficient of variation, which is a measure of the variability in reference genome length associated with each HMM. For each HMM on a viral contig, CheckV identifies the range of completeness values corresponding to the fifth and 95th percentiles of the distribution of reference genome length containing the same HMM (for example, 35–65% completeness). In theory, we expect the true completeness to be greater than the lower bound 95% of the time, below the upper bound 95% of the time and between both bounds 90% of the time. In practice, however, these outcomes are less frequent due to error in the underlying estimates. CheckV performs this step for each HMM, resulting in a distribution of completeness ranges for each contig (for example, 45–67, 35–55 and 42–49%). Finally, CheckV takes a weighted average of the ranges, where the weights are equal to the inverse of the coefficient of variation with a maximum value of 50. Therefore, HMMs with a low coefficient of variation (which are associated with genomes of consistent length) receive higher weight.

### Confidence levels for AAI-based completeness estimates

We conducted a large-scale benchmarking experiment to derive confidence levels for AAI-based completeness estimation. First, we extracted a random fragment from each of CheckV’s reference genomes to simulate metagenomic contigs of varying length (200 and 500 bp and 1, 2, 5, 10, 20 and 50 kb). Next, we used CheckV to compute the alignment score between each contig and each complete genome in the reference database. We then compared the true genome length of each contig (that is, the length before fragmentation), *L*, to the estimated genome length based on each matched reference genome, ${\hat{\it L}}$, and computed the relative unsigned error, as $100 \times \left| {{\it{L}} - {\hat{\it L}}} \right|/{\it L}$. We then computed the median relative unsigned error after grouping the estimates based on their alignment score and contig length. Finally, we determined three confidence levels: high confidence (0–5% median unsigned error), medium confidence (5–10% median unsigned error) and low confidence (>10% median unsigned error). Using this information, CheckV reports a confidence level in the estimated completeness value for each input contig based on contig length and alignment score (that is, a combination of AAI and AF) to the top database hit. By default, only medium- and high-confidence estimates are included in the final report.

### Database of complete viral genomes for AAI-based completeness estimation

We downloaded 30,903 genomes from NCBI GenBank on 1 June 2019, excluding 1,937 that were indicated as ‘partial’, ‘chimeric’ or ‘contaminated’. Of the remaining 28,966, 677 (2.3%) were labeled as ‘metagenomic’ or ‘environmental’, suggesting that the vast majority are derived from cultivated isolates.

Next, we used CheckV to systematically search for complete genomes of uncultivated viruses from publicly available and previously assembled metagenomes, metatranscriptomes and metaviromes. An assembled contig was considered complete if it was at least 2,000 bp in length and included a DTR of at least 20 bp (DTR contigs). We searched for DTR contigs in the following datasets: 19,483 metagenomes and metatranscriptomes from IMG/M (accessed September 2019)^35^, 11,752 metagenomes from MGnify (accessed 16 April 2019)^36^, 9,428 metagenomes assembled by Pasolli et al.^38^, an expanded collection of 4,763 metagenomes from the HGM dataset^37^, 1,831 viromes from HuVirDB^39^ and 145 viromes from the Global Ocean Virome 2.0 dataset^6^.

From this initial search, we identified a total of 751,567 DTR contigs. To minimize false positives and other artifacts, we removed the following: (1) 45,448 contigs with low-complexity repeats (for example, AAAAA…), as determined by dustmasker from the BLAST+ package v.2.9.0 (ref. ^56^); (2) 11,359 contigs classified as proviral by CheckV; (3) 5,737 contigs with repeats occurring more than five times per contig, which could represent repetitive genetic elements such as clustered regularly interspaced short palindromic repeat (CRISPR) arrays; (4) 6,543 contigs that contained a large duplicated region spanning ≥20% of the contig length, resulting from rare instances where assemblers concatenate multiple copies of the same genome; and (5) 1,293 contigs containing ≥1% ambiguous base calls. After application of these filters, 686,030 contigs remained (91.3% of the total).

Next, we used a combination of CheckV marker genes and VirFinder^9^ to classify 116,666 DTR contigs as viral. First, the DTR contigs were used as input to VirFinder v.1.1 with default parameters, and to CheckV to identify viral and microbial marker genes. We additionally searched for genes related to plasmids and other nonviral mobile genetic elements using a database of 141 HMMs from recent publications^57–59^. A contig was classified as viral if the number of viral genes exceeded that of microbial and plasmid genes (*n* = 99,345), or VirFinder reported a *P* < 0.01 with no plasmid genes and no more than one identified microbial gene (*n* = 36,084).

### Taxonomic annotation of CheckV reference genomes

Annotations were determined based on HMM searches against a custom database of 1,000 taxonomically informative HMMs from the VOG database (http://vogdb.org/). These HMMs were selected for major bacterial and archaeal viral groups with consistent genome length and at least ten representative genomes, including: *Caudovirales*, CRESS-DNA and *Parvoviridae*, *Autolykiviridae*, *Fusello-* and *Guttaviridae*, *Inoviridae*, *Ligamenvirales Ampulla- Bicauda-* and *Turriviridae*, *Microviridae* and *Riboviria*. For each group, VOGs found in ≥10% of the group members and never detected outside of this group were considered as marker genes. All CheckV reference genomes were annotated based on the clade with the most HMM hits. Overall, 96.4% of HMM hits were to a single viral taxon.

### Validating the completeness of CheckV reference genomes

Next, we validated the completeness for all GenBank genomes and DTR contigs. First, we used CheckV to estimate the completeness for all sequences after excludsion of self-matches. This was performed using a database of GenBank sequences only and another of DTR contigs only. Any sequence with <90% estimated completeness using either database was excluded (medium- and high-confidence estimates only). Second, we compared genome length to the known distribution of genome length for the annotated viral taxon (for example, *Microviridae*). Any genome considered an outlier or shorter than the shortest reference genome for the annotated clade was excluded. After application of these exclusion filters, we then selected genomes for inclusion with ≥90% estimated completeness using either database (medium- and high-confidence estimates only) or >30 kb without a completeness estimate. These selection criteria were chosen to minimize the number of false positives (that is, genome fragments wrongly considered complete genomes) at the cost of some false negatives (that is, removal of truly complete genomes). This resulted in 24,834 GenBank genomes and 76,262 DTR contigs that were used to form the final CheckV genome database.

### Generating a nonredundant set of CheckV reference genomes

Average nucleotide identity (ANI) and alignment fraction (AF) were computed between the 24,834 GenBank genomes and 76,262 DTR contigs using a custom script. Specifically, we used blastn from the BLAST+ package v.2.9.0 (option: perc_identity=90 max_target_seqs=10000) to generate local alignments between all pairs of genomes. Based on this, we estimated ANI as the average DNA identity across alignments after weighting the alignments by length. The AF was computed by taking the total length of merged alignment coordinates and dividing this by the length of each genome. Clustering was then performed using a greedy, centroid-based algorithm in which (1) genomes were sorted by length, (2) the longest genome was designated as the centroid of a new cluster, (3) all genomes within 95% ANI and 85% AF were assigned to that cluster and (4) steps 2 and 3 were repeated until all genomes had been assigned to a cluster, resulting in 52,141 nonredundant genomes.

### Benchmarking estimation of genome completeness

To benchmark genome completeness estimates, we used 2,000 uncultivated, complete viral genomes from IMG/VR (>20-bp DTR). We used IMG/VR genomes, because these are derived from diverse habitats and represent highly novel sequences. After removal of terminal repeats, a single genome fragment was randomly extracted from each IMG/VR genome (1–100% completeness). These sequences were used as input to CheckV, VIBRANT v.1.2.0 (ref. ^11^) and viralComplete^22^. For CheckV we used the flag ‘--max_aai 95’ to exclude closely related genomes in the CheckV database. For VIBRANT, we used the flag ‘--virome’ to increase sensitivity. For viralComplete, completeness was determined based on the ratio of contig length to that of the corresponding genome from NCBI RefSeq. Completeness estimates >100% were set to 100%. Additionally, we benchmarked CheckV using genome fragments derived from NCBI Genbank genomes and used the flag ‘--max_aai 95’ to exclude closely related genomes in the CheckV database.

### Benchmarking detection of host regions on proviruses

To benchmark CheckV’s detection of host regions, we constructed a mock dataset of proviruses: 382 viral genomes were downloaded from NCBI GenBank (after 1 June 2019) and paired with 76 GTDB genomes (71 bacterial, 5 archaeal). None of the 382 genomes were used to train CheckV (that is, selection of HMMs and bit-score thresholds). The pairing was performed at the genus level based on the annotated names of virus and host (for example, *Escherichia* phage paired with *Escherichia* bacterial genome). When multiple GTDB genomes were available for a given bacterial genus, we chose that with the highest CheckM quality score and selected a maximum of ten GenBank genomes per bacterial genus to reduce the influence of a few over-represented groups. Any GenBank or GTDB genome that was used at any stage for training CheckV was excluded. Proviruses were simulated at varying contig lengths (5, 10, 20, 50 and 100 kb) with varying levels of host contamination (10, 20 and 50%; defined as the percentage of contig length derived from the microbial genome). Microbial genome fragments were appended to either the 5' or 3' end of the viral fragment at random. As a negative control, we also simulated contigs that were entirely viral (that is, no flanking microbial region) at the same contig lengths.

Mock proviruses were used as input to CheckV using default parameters. For comparison, we also ran VIBRANT v.1.2.0 (ref. ^11^), VirSorter v.1.0.5 (ref. ^10^), PhiSpy v.3.7.8 (ref. ^23^) and Phigaro v.2.2.5 (ref. ^24^). All tools were run with default options with the exception of VIBRANT and VirSorter, which were run with the flag ‘--virome’ to increase sensitivity. Nucleotide sequences were used as input to all tools, except PhiSpy, for which we first ran Prokka v.1.14.5 (ref. ^60^) to generate the required input file. A contig was classified as a provirus if it contained a predicted viral region covering <95% of its length. Each prediction was then classified as a true positive (provirus classified as provirus), false positive (viral contig classified as provirus), true negative (viral contig not classified as provirus) or false negative (provirus classified as provirus). For the true positives, we also compared the true and predicted lengths of the host region.

### Application of CheckV to diverse viral genome collections

We downloaded 735,106 contigs >5 kb from IMG/VR 2.0 (ref. ^25^), after exclusion of viral genomes from cultivated isolates and proviruses identified from microbial genomes. We also downloaded 488,131 contigs >5 kb or circular from the GOV 2.0 dataset^6^ (datacommons.cyverse.org/browse/iplant/home/shared/iVirus/GOV2.0). These were used as input to CheckV to estimate the completeness, identify host–virus boundaries and predict closed genomes. When running the completeness module, we excluded perfect matches (100% AAI and 100% AF) to prevent any DTR contig from matching itself in the database (since IMG/VR 2.0 and GOV 2.0 were used as data sources to form the CheckV database). A Circos plot^61^ was used to link IMG/VR contigs to their top matches in the CheckV database. Protein-coding genes were predicted from proviruses using Prodigal and compared to HMMs from KEGG Orthology (release 2 October 2019)^45^ using hmmsearch from the HMMER package v.3.1b2 (≤1 × 10^–5^ and score ≥30). Pfam domains with the keyword ‘integrase’ and ‘recombinase’ were also identified across all proviruses.

The largest DTR contig we identified from IMG/VR was further annotated to illustrate the type of virus and genome organization represented (IMG ID: 3300025697_____Ga0208769_1000001). Coding sequence prediction and functional annotations were obtained from IMG^35^. Annotation for virus hallmark genes including a terminase large subunit (TerL) and major capsid protein were confirmed via HHPred v.3.2.0 (ref. ^62^) (databases included PDB 70_8, SCOPe70 2.07, Pfam-A 32.0 and CDD 3.18, score >98). A circular genome map was drawn with CGView^63^. To place this contig in an evolutionary context, we built a TerL phylogeny including the most closely related sequences from a global search for large phages^42^. The TerL amino acid sequence from the DTR contig was compared to all TerL sequences from the ‘huge phage’ dataset via blastp (≤1 × 10^–5^, score ≥50) to identify the 30 most similar sequences (sorted based on blastp bit-score). These reference sequences and DTR contigs were aligned with MAFFT v.7.407 (ref. ^64^) using default parameters, the alignment automatically cleaned with trimAL v.1.4.rev15 with the option ‘--gappyout’^65^ and a phylogeny built with IQ-Tree v.1.5.5, with default model selection (optimal model suggested: LG+R4)^66^. The resulting tree was visualized with iToL^67^.

### Reporting Summary

Further information on research design is available in the Nature Research Reporting Summary linked to this article.

## Online content

Any methods, additional references, Nature Research reporting summaries, source data, extended data, supplementary information, acknowledgements, peer review information; details of author contributions and competing interests; and statements of data and code availability are available at 10.1038/s41587-020-00774-7.

## Supplementary information

Supplementary Information Supplementary Text.

Reporting Summary

Supplementary Software 1. CheckV software package.

Supplementary Tables 1–12.

## Data Availability

The complete CheckV database, including HMMs, GenBank genomes and DTR contigs, is available at https://portal.nersc.gov/CheckV/.

## Code availability

CheckV is written in Python and is freely available as open source software at https://bitbucket.org/berkeleylab/CheckV under a BSD license.
