# Supplementary material for: CheckV assesses the quality and completeness of metagenome-assembled viral genomes
Source: Nat Biotechnol. 2020 Dec 21;39(5):578–85. doi: 10.1038/s41587-020-00774-7 (PMC8116208; doi:10.1038/s41587-020-00774-7)
Supplement: Supplementary file 1 — Supplementary Text. [file 41587_2020_774_MOESM1_ESM.pdf]

nature research
https://doi.org/10.1038/s41587-020-00774-7

Supplementary information

CheckV assesses the quality and completeness of metagenome-assembled viral genomes

In the format provided by the authors and unedited

Supplementary text

Investigating DTR contigs classified as *Retrovirales* and *Riboviria*

Since genomes from *Retrovirales* and *Riboviria* (i.e. RNA viruses) are typically linear, we further analyzed DTR sequences affiliated to these clades to identify putative errors or misannotation. For *Retrovirales*, most sequences with DTR (>97%) were ≤ 15kb, which is consistent with the size range of complete retrovirus genomes. A best blast hit affiliation of these contigs against NCBI Viral RefSeq revealed that the vast majority (>90%) were most similar to *Metaviridae*, i.e. retrotransposon-like with long terminal repeats. The second most common group to which these sequences were affiliated was the *Caulimoviridae* family, with a circular genome. Hence, DTR contigs affiliated to *Retrovirales* seemingly represented genuine complete viral genomes and/or retrotransposons.

For *Riboviria*, >97% of the DTR contigs were ≤15kb, which is a plausible size for complete RNA virus genomes. A more detailed gene annotation of the 101 representatives contigs for these DTR sequences affiliated to *Riboviria* revealed 3 main groups. First, 68 contigs encoded an RdRP where the closest relative in NCBI Viral RefSeq was found within the Narna-like clade. Genomes from this RNA virus group, which includes mitoviruses, were previously observed to assemble as circular contig, likely either because of the existence of a circular form of the genome or because of a replication mechanism involving a concatemer intermediary [1, 2]. These contigs, which represent the majority of the set, thus likely represent genuine complete *Riboviria* genomes. Another set of 15 sequences lacked an RdRP or other clear taxonomic marker gene but shared similarity to uncharacterized genes in known *Riboviria* genomes. The last set of 18 DTR contigs could be identified as members of the CRESS-DNA group (i.e. ssDNA viruses), based on the presence of a replication-associated gene typical from this group. These sequences represent complete genomes but were mis-affiliated as *Riboviria* instead of CRESS-DNA and were therefore excluded from Figure 2B and Figure 2C.

Estimating completeness for giant virus MAGs and NCLDV isolate virus genomes

We performed two experiments to assess whether CheckV was suitable for estimating the completeness of giant viruses. First, we applied the CheckV to 2,074 giant virus metagenome-assembled genomes (GVMAGs) described in Schulz et al. [3]. Prior to running CheckV, NCLDV genomes that consisted of multiple contigs were concatenated to a single contig. CheckV completeness estimates for GVMAGs were then compared to previously determined completeness estimates based on low-copy number nucleocytoplasmic virus orthologous groups (NCVOGs) [4] described in Schulz et al. [3]. CheckV completeness estimates were correlated with the Schulz et al. estimates, particularly for high-confidence CheckV estimates (Figure S4A). These correlations imply that CheckV gave broadly similar results compared to Schulz et al. and may be suitable to evaluate the completeness of some metagenome assembled NCLDV genomes.

To directly assess CheckV's accuracy for giant viruses, we applied it to 182 nucleocytoplasmic large DNA virus (NCLDV) isolate genomes (Figure S4B). CheckV showed good performance and obtained completeness estimates for 180/182 NCLDV genomes (98.9%) with 158/180 complete genomes having >90% estimated completeness (87.7%). However, many of the NCLDV genomes were included in the CheckV database. To exclude CheckV reference genomes closely related to the published NCLDV isolate genomes, we used the --max_aai flag with different AAI cutoff values (50, 75, 90, 100). As expected, CheckV performance decreased for more distantly related NCLDV genomes (Figure S4B). For example, after excluding CheckV reference genomes that had an AAI similarity of 50% or greater, CheckV obtained completeness estimates for 142/182 NCLDV genomes (78.0%) with 97/142 complete genomes having >90% estimated completeness (68.3%).

Additional analysis of the 528 kb viral contig from Ace Lake in Antarctica

The IMG/VR contig (IMG contig ID: Ga0222679_1000001) was identified from an Ace lake, Antarctica sample (IMG taxon ID: 3300022858) and predicted as complete based on the presence of a 127-bp DTR (Figure S5A). The terminal repeat did not contain any low complexity regions and occurred three times on the contig (twice at termini and one other time). The contig was classified as viral based on a VirFinder p-value of 0.010 and score of 0.92 as well as the presence of 35 CheckV viral markers of 601 total protein-coding genes. Manual annotation also revealed the presence of a phage-like terminase large subunit (TerL) and a major capsid protein, two hallmark genes of phages in the *Caudovirales* order. 19 CheckV microbial markers were found, but these were interspersed between viral genes and did not result in CheckV predicting any host regions. A self-alignment of the contig with blastn did not reveal any large duplicated regions beyond the 127-bp DTR.

To validate circularity, we first ran CheckV and obtained an estimated completeness of 100%. The completeness estimate was based on a 100% ANI / 99.8% AF match to a CheckV sequence (DTR_285855) that was derived from a different sample from the same lake (IMG taxon ID: 3300025697, IMG contig ID: Ga0208769_1000001). As further validation, we performed read mapping from the sample (sequencing project ID: 1166905) to the 528,258 bp circular contig in order to test whether any reads spanned the circular breakpoint. After mapping with Bowtie 2 [5] using default options, we discarded paired end reads with more than 2 mismatches and discarded reads mapped to the same strand. After these filters 107,332 reads were mapped to the contig with a median insert length of 311 bp and read length of 150 bp. Supporting the circularity, we identified 10 reads with an insert length of 528,046 bp that spanned nearly the entire contig; assuming these reads instead spanned the circular breakpoint, then their insert lengths would instead be 212 bp, which is plausible for this dataset.

While *Caudovirales* genomes are typically ~50kb, larger genomes of ~500kb have been reported [6]. Recently, a set of new large (≥200kb) phages were reported from metagenome assemblies from which 10 major clades were proposed [7]. Based on a TerL phylogeny, contig Ga0208769_1000001 seems to be a new virus related to one of these clades ("Biggiephage", Figure S5B). Several members of the Biggiephage clade encode CRISPR arrays [7], and similarly contig Ga0208769_1000001 encodes a Type I-C-like

CRISPR array (Figure S5A). No host could be predicted for Ga0208769_1000001 based on a search against the IMG CRISPR spacer database. Similarly, no significant match was identified between the spacers encoded on contig Ga0208769_1000001 and other Ace Lake contigs, hence it is unclear at this stage which elements are targeted by this CRISPR array. Finally, contig Ga0208769_1000001 included an unusually high number of transposases (14) distributed throughout the sequence, which suggests that mobile genetic elements may play a role in the large size of this genome.

Supplementary references

1. Bruenn, J.A., B.E. Warner, and P. Yerramsetty, *Widespread mitovirus sequences in plant genomes.* PeerJ, 2015. **3**: p. e876.
2. Hintz, W.E., et al., *Two novel mitoviruses from a Canadian isolate of the Dutch elm pathogen Ophiostoma novo-ulmi (93-1224).* Virol J, 2013. **10**: p. 252.
3. Schulz, F., et al., *Giant virus diversity and host interactions through global metagenomics.* Nature, 2020. **578**(7795): p. 432-436.
4. Yutin, N., et al., *Eukaryotic large nucleo-cytoplasmic DNA viruses: clusters of orthologous genes and reconstruction of viral genome evolution.* Virol J, 2009. **6**: p. 223.
5. Pongor, L.S., R. Vera, and B. Ligeti, *Fast and sensitive alignment of microbial whole genome sequencing reads to large sequence datasets on a desktop PC: application to metagenomic datasets and pathogen identification.* PLoS One, 2014. **9**(7): p. e103441.
6. Mendoza, S.D., et al., *A bacteriophage nucleus-like compartment shields DNA from CRISPR nucleases.* Nature, 2019.
7. Al-Shayeb, B., et al., *Clades of huge phages from across Earth's ecosystems.* Nature, 2020. **578**(7795): p. 425-431.
