# Supplementary figures and images for: CheckV assesses the quality and completeness of metagenome-assembled viral genomes
Source: Nat Biotechnol. 2020 Dec 21;39(5):578–85. doi: 10.1038/s41587-020-00774-7 (PMC8116208; doi:10.1038/s41587-020-00774-7)

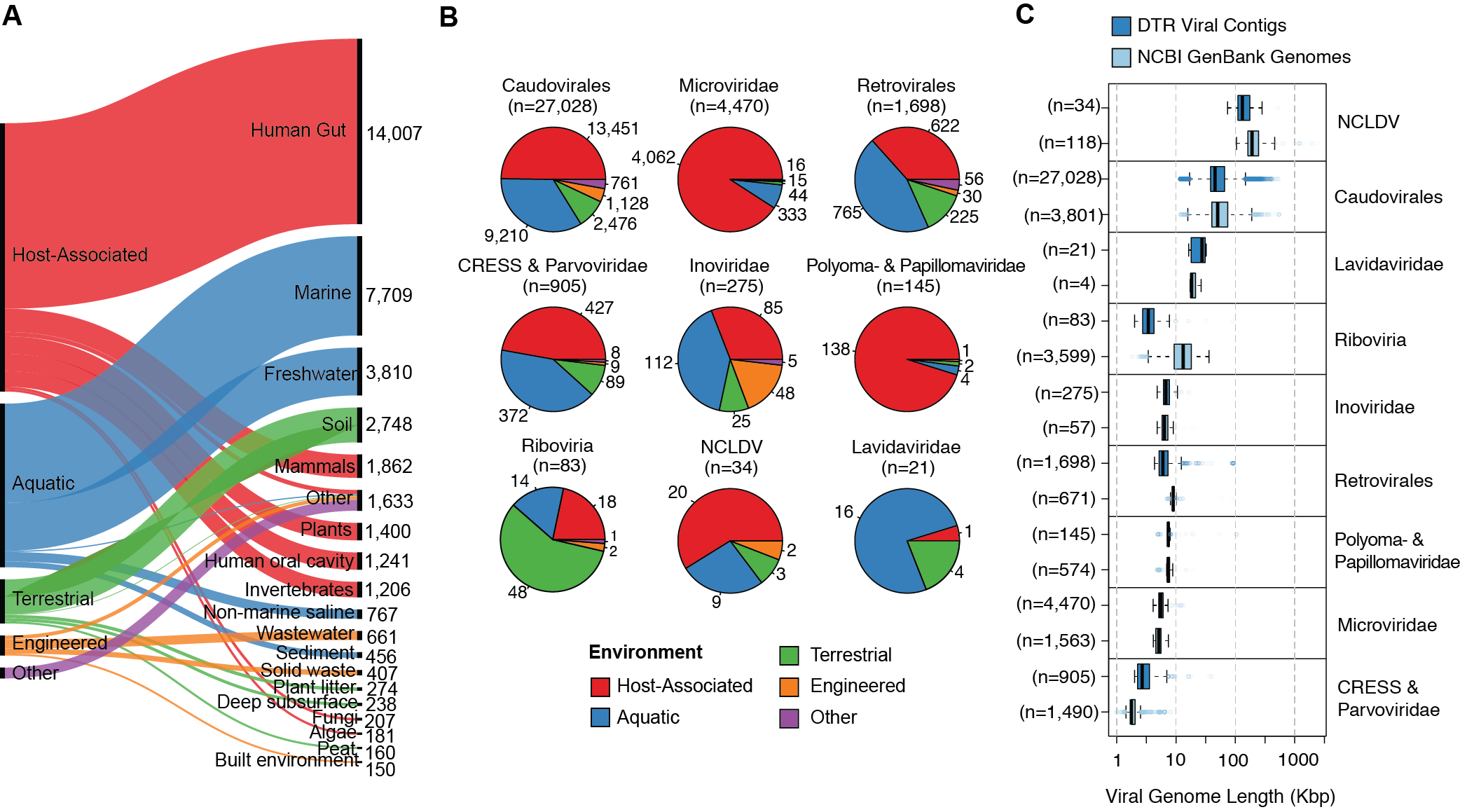

Supplement: Supplementary file 3 — CheckV software package. [file 41587_2020_774_MOESM3_ESM.zip › berkeleylab-checkv-95e3ee217c91/database.png]

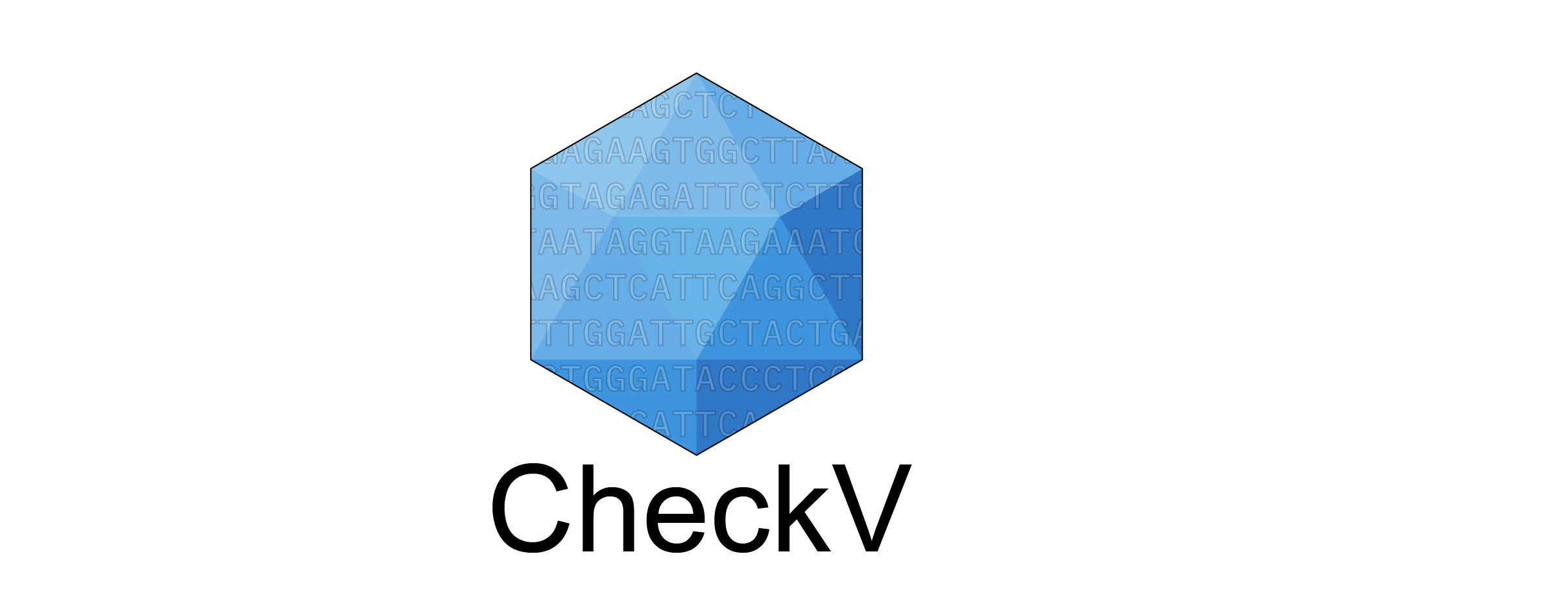

Supplement: Supplementary file 3 — CheckV software package. [file 41587_2020_774_MOESM3_ESM.zip › berkeleylab-checkv-95e3ee217c91/logo.png]

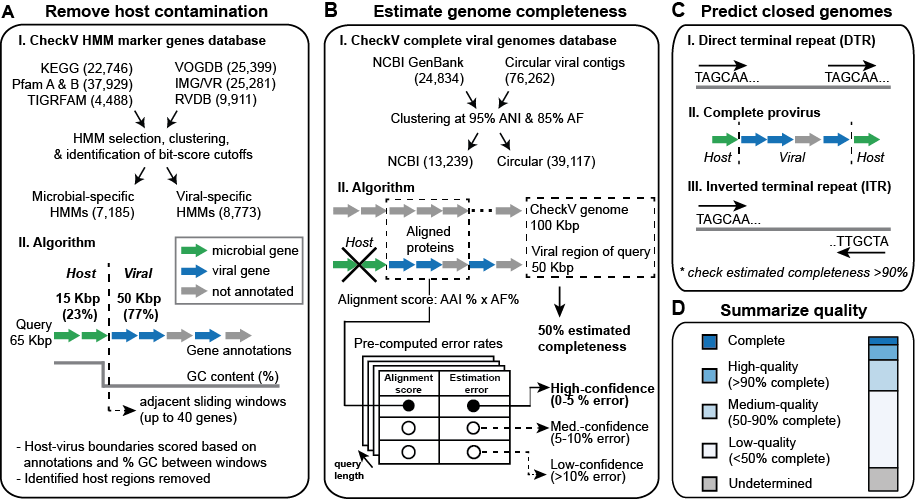

Supplement: Supplementary file 3 — CheckV software package. [file 41587_2020_774_MOESM3_ESM.zip › berkeleylab-checkv-95e3ee217c91/pipeline.png]
